# Preventive Dentistry

**Published:** 1916-06

**Authors:** W. Irving Carlsen

**Affiliations:** Chicago, Ill.


### PREVENTIVE DENTISTRY.

BY W. IRVING CARLSEN, D. D. S., CHICAGO, ILL.

Preventive dentistry seems now to be a nation-wide watchword even to the extent that many public-spirited individuals have taken the initiative and brought before their communities men of national reputation to give instructive talks. This puts the dentist in a position wherein he must of necessity become a great community factor.

In the past decade or two much effort has been brought forth to perfect means to prevent caries and other pathological conditions of the teeth and surrounding organs. There can be no question of a doubt but that oral prophylaxis represents the highest development in dental science. We have long been taught that a clean tooth will not decay and I believe that the same rule holds true that a clean tooth kept clean will not become affected with pyorrhea.

We now come to the point to decide what a clean tooth really is. A tooth which has had a cursory brushing with pumice stone is manifestly clean, but its state of cleanliness is of but short duration. Such cleaning done once or twice a year will not keep the mouth in a prophylactic condition. A clean tooth, and I mean a tooth which is as surgically clean as it is possible to make it, must of necessity be one which has had all of its scratches and pits eradicated and then be brilliantly polished.

This process may seem at first sight to be very elaborate and tedious, but on close analysis we find it to be a very desirable one from all angles. From the patient's standpoint the ultimate result is the one to be sought. Therefore, for this one reason it seems altogether logical to me that the placing of a mouth in such condition as to reduce the possibility of caries and pyorrhea to a minimum is a true service giving procedure.

This process of placing the mouth in a prophylactic condition by means of extensive tooth polishing can be brought about in the following manner: With the gem points or other composition stones of fine grit the tooth should be ground until the grosser ridges, grooves and pits have been removed. If the stone be kept cool with a stream of water, this operation will not be painful. Arkansas stones are then used to complete the grinding operations. The interproximal surfaces of the teeth can be planed with interproximal trimmers and smoothed down with sandpaper strips.

Now we come to the root of the tooth and the methods of polishing it. With scaling instruments the root must be planed smooth of all its pericemental pits and grooves. It is of extreme importance that these be removed, for, microscopical as they are, they are tiny culture beds for pyogenic organisms. One should take particular care in this part of the operation to use a scaling instrument that will not leave scratches and grooves on the root.

After the root has been thoroughly planed and smoothed, the enamel shoulder just under the gingivae should be carefully rounded. Nature will not tolerate any sharpness or roughness and therefore because the sharp enamel shoulder is irritating to the delicate gum tissue it should be smoothed.

We will now thoroughly disk the crown of the tooth, using first a coarse sandpaper disk and ending with cuttle fish disks. In this process all of the tiny scratches which were left from the stones are removed. This will leave the tooth in a smooth but dull condition. A very high and lastingly brilliant polish can be put on the tooth by first using a moosehide wheel charged with silex and finishing with a rubber cup charged with whiting and oxid of tin. Wide polishing tape charged with these powders will effectively polish the proximal surfaces of the teeth. The necks of the teeth must receive considerable attention and should be polished with an orange wood point and silex. For this purpose an engine porte polisher is a very efficient instrument.

These are the salient features in the process of polishing teeth. To sum up the whole, we find that the practice of oral prophylaxis is remunerative, and with the co-operation of the patient in the daily cleansing of the teeth and surrounding structures, we can do much toward the actual prevention of many obscure and seemingly incurable diseases to which the human flesh is heir.—*Review.*

